# Predicting climate-driven distribution shifts in *Hyalomma marginatum* (Ixodidae)

**DOI:** 10.1017/S0031182023000689

**Published:** 2023-09

**Authors:** Olcay Hekimoglu, Can Elverici, Arda Cem Kuyucu

**Affiliations:** 1 Biology Department, Hacettepe University, Ankara, Turkey; 2 Biodiversity Institute, University of Kansas, Lawrence, KS, USA

**Keywords:** climate change, Crimean–Congo haemorrhagic fever, ecological niche modelling, *Hyalomma marginatum*, MaxEnt, ticks

## Abstract

*Hyalomma marginatum* is an important tick species which is the main vector of Crimean–Congo haemorrhagic fever and spotted fever. The species is predominantly distributed in parts of southern Europe, North Africa and West Asia. However, due to ongoing climate change and increasing reports of *H. marginatum* in central and northern Europe, the expansion of this range poses a potential future risk. In this study, an ecological niche modelling approach to model the current and future climatic suitability of *H. marginatum* was followed. Using high-resolution climatic variables from the Chelsa dataset and an updated list of locations for *H. marginatum*, ecological niche models were constructed under current environmental conditions using MaxEnt for both current conditions and future projections under the ssp370 and ssp585 scenarios. Models show that the climatically suitable region for *H. marginatum* matches the current distributional area in the Mediterranean basin and West Asia. When applied to future projections, the models suggest a considerable expansion of *H. marginatum*'s range in the north in Europe as a result of rising temperatures. However, a decline in central Anatolia is also predicted, potentially due to the exacerbation of drought conditions in that region.

## Graphical abstract

**Figure:**
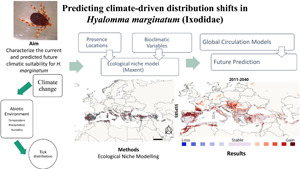

## Introduction

Human intervention in ecosystems, including globalization, urbanization, rapid transportation, land use and climate change, has led to a global increase in the range, distribution and transmission rate of numerous pathogens and vector organisms that serve as reservoirs for these pathogens (Kovats *et al*., 2001; Andersen and Davis, 2017; Carvalho *et al*., 2017; Semenza and Suk, 2018; Aguilar-Domínguez *et al*., 2021; Leder *et al*., 2021). While the most severe impacts of climate change are expected to affect ectotherm populations in the tropics, population growth rates of insects and other arthropods are also anticipated to increase in mid-to-high latitude regions due to the influence of warmer temperatures on growth rates (Deutsch *et al*., 2008; Bonebrake and Deutsch, 2012; Rocklöv and Dubrow, 2020). In addition to the effects of temperature on the biology of disease-transmitting vector organisms, increased precipitation and extreme precipitation events in some regions may lead to an increase in vector abundance, whereas longer dry periods might increase tick mortality (Githeko *et al*., 2000; Rocklöv and Dubrow, 2020).

Ticks are the vectors of numerous critical pathogens and pose a significant threat to animal and public health (Jongejan and Uilenberg, 2004). The species *Hyalomma marginatum* Koch, 1844 presents a substantial health threat as the main vector of Crimean–Congo haemorrhagic fever (CCHF) and also the vector of babesiosis and Rickettsia in the Mediterranean region, Africa and Asia (Ergönül, 2006; Vatansever *et al*., 2007; Gale *et al*., 2010; Ros-García *et al*., 2011; Bonnet *et al*., 2022; Sultankulova *et al*., 2022). Effective and sustainable control of ticks, like many arthropod pests and vectors, can be achieved with a solid understanding of the species' biology, ecology and distribution (Bonnet *et al*., 2022). Knowledge of *H. marginatum*'s current distribution is crucial for projecting areas at risk for CCHF expansion. The Mediterranean basin holds special importance as the main distributional area and reservoir for *H. marginatum*. The Mediterranean is also among the areas projected to be most negatively affected by climate change (Ulbrich *et al*., 2006; Bardsley and Edwards-Jones, 2007; Newbold *et al*., 2020). Studies have indicated an increase in CCHF transmission in the Mediterranean basin (Maltezou and Papa, 2010) and a tendency for the northward expansion of CCHF's range in this region (Estrada-Peña and Venzal, 2007; Williams *et al*., 2015; Andersen and Davis, 2017; Fernández-Ruiz and Estrada-Peña, 2021). Recent reports have shown that the vector of CCHF, *H. marginatum* Koch, 1844 might have expanded its geographic range and shifted northwards into previously unoccupied areas (Vial *et al*., 2016; Bah *et al*., 2022). Furthermore, the expansion of suitable areas has been documented in central Europe and Balkans in addition to Mediterranean countries (Fernández-Ruiz and Estrada-Peña, 2021). Recent detections have highlighted that northern latitudes might be becoming more suitable for *H. marginatum* activity and survival as a result of climate change; however, most of these are records of individual tick detections and yet there is not sufficient evidence of establishment of northern populations (Duscher *et al*., 2018; Chitimia-Dobler *et al*., 2019; Grandi *et al*., 2020; McGinley *et al*., 2021).

Thanks to advances in machine-learning algorithms in ecology, such as MaxEnt (Phillips and Schapire, 2004; Elith *et al*., 2011; Crisci *et al*., 2012; Merow *et al*., 2013) and the advent of high-resolution climatic datasets such as Chelsa (Karger *et al*., 2017) and WorldClim (Fick and Hijmans, 2017), ecological niche modeling ENM has become a fundamental method for investigating possible current and future distributions of disease-transmitting vectors over the past 2 decades (Chalghaf *et al*., 2018; Raghavan *et al*., 2020; Aguilar-Domínguez *et al*., 2021; Alkishe *et al*., 2021; Moo-Llanes *et al*., 2021). Correlative ecological niche models primarily rely on combining georeferenced species records with predictive environmental variables (e.g. climatic, topographic, soil) to build a coefficient matrix representing the organism's multidimensional niche (Warren and Seifert, 2011; Peterson and Soberón, 2012; Sillero and Barbosa, 2021). ENMs have been widely used to predict the potential future distributions of vectors under various global circulation scenarios of climate, in addition to their current potential niche (Aguilar-Domínguez *et al*., 2021; Alkishe and Peterson, 2022; Wu *et al*., 2022).

The main objective of this study was to predict climatically suitable areas for *H. marginatum* under both present-day conditions and future climate scenarios. To achieve this aim, bioclimatic parameters for present and near-future scenarios (2011–2040 and 2041–2070) obtained from the Chelsa database and *H. marginatum* locations from the literature and previous field surveys by the authors were used to build ecological niche models with MaxEnt to show suitable areas for present and future projections. The findings of this study will help predict potential new suitable areas in the Mediterranean basin and Europe that *H. marginatum* may colonize in the future and enhance surveillance efforts in areas identified as high risk.

## Materials and methods

### Occurrence records

Local data from Turkey were derived from 2 sources: (1) from published studies by first author (O. H.) until 2021, which used both morphological and molecular methods to identify tick samples (Hekimoglu and Ozer, 2017; Hekimoglu *et al*., 2021) and additional field collected samples from 2018 (*n* = 13 locations) and 2021 (*n* = 7 locations) which have been identified using 16s rDNA. Morphological identification was conducted using taxonomic keys (Apanaskevich and Horak, 2008; Estrada-Peña *et al*., 2018). Since *H. marginatum* populations show morphological variations (Apanaskevich and Horak, 2008) and *Hyalomma* species have been one of the most misidentified taxa using morphological methods (Estrada-Pena and De La Fuente, 2016), conducting a molecular step is extremely important. For this purpose, mitochondrial 16S rDNA sequences (Mangold *et al*., 1998) were generated for samples of localities where molecular data were not generated before. In total, 66 geographical points from Turkey were included in the analyses.

To represent the distribution area of the species worldwide, we included geographic locations of *H. marginatum* from different parts of the world from a previous compilation by Estrada-Pena and De La Fuente (2016), composed of literature reviews between 1970 and 2014. Firstly, the geographic information of *H. marginatum* was recorded on a separate data sheet. Secondly, these raw records were cleaned and reduced by removing localities, (1) where tick collection had been conducted from birds since finding ticks on birds does not mean that ticks can establish populations in these areas (Estrada-Peña *et al*., 2011; Fernández-Ruiz and Estrada-Peña, 2021); and (2) sampling information from Cyprus was removed as molecular data do not confirm that *H. marginatum* exists in Cyprus (Hekimoglu and Ozer, 2017). After these steps, a total of 565 geographic coordinates were obtained.

### Environmental variables

For environmental predictors, Chelsa V2.1 dataset was used (Karger *et al*., 2017, 2022) which is available at https://chelsa-climate.org, a relatively new high-resolution (30 arcsec, ~1 km) climate dataset that includes additional important microclimatic variables in addition to the counterparts (Chelsa-Bioclim) of popular WorldClim bioclim variables (Hijmans *et al*., 2005; Fick and Hijmans, 2017). These include variables related to microclimate, water content of air and humidity which are important for *H. marginatum* (Estrada-Peña *et al*., 2011; Estrada-Peña, 2023). Furthermore, while the WorldClim dataset used for current distribution predictions uses extrapolations made between 1970 and 2000, the Chelsa dataset uses a dataset created for the period between 1980 and 2010. The additional important variables (Chelsa-Bioclim+) included near surface relative humidity (hurs), vapour pressure deficit (vpd), climate moisture index (cmi), growth degree days above 0°C, net primary productivity (npp) and surface downwelling shortwave radiation (rsds). Growth season temperature (gst) and growth season precipitation (gsp) were also included, which are indicative of *H. marginatum*, whose activity coincides greatly with the growth season (Estrada-Peña *et al*., 2011). Bioclim variables 8, 9, 18 and 19 from the Worldclim dataset (Fick and Hijmans, 2017) are reported to have significant spatial artefact problems visible as anomalous discontinuities between neighbouring pixels and thus it is generally advised to remove them before carrying out analyses (Escobar *et al*., 2014; Escobar, 2020; Aguilar-Domínguez *et al*., 2021; Alkishe and Peterson, 2022). In this study, these variables were removed from the Chelsa dataset since it was observed that the same artefacts are also present in Chelsa-Bioclim data. All remaining variable rasters were clipped to the area of interest, which is between latitudes −20° and 60° and longitudes 20° and 60° WGS84. All geographic computations were carried out with QGIS (QGIS Geographic Information System, 2022), GDAL library in Python (Open Source Geospatial Foundation, 2022) and R version 4.2.2 (R Core Team, 2022).

### Future projections

For future predictions, environmental variables from different global circulation models (GCM) of ssp370 and ssp585 scenarios for CMIP6 (Eyring *et al*., 2016) were used. ‘Shared socioeconomic pathways’ (SSPs) complement the ‘representative concentration pathways’ (RCPs) by involving socio-economic factors such as population growth, urbanization and technological advances, ssp585 represents the worst-case scenario while ssp370 represents a middle way between the worst and more optimistic scenarios (Hausfather, 2020). These bad-to-worst case scenarios were chosen for a more prudent risk prediction as anthropogenic climate risks are becoming much more difficult to manage and complicated all around the world (Kemp *et al*., 2022). All of the available scenarios for Chelsa dataset were included for a better estimation. These include UKESM1-0-LL (Sellar *et al*., 2019), MRI-ESM2-0 (Oshima *et al*., 2020), MPI-ESM1-2 (Mauritsen *et al*., 2019), IPSL-CM6-LR (Boucher *et al*., 2020) and GFDL-ESM4 (Dunne *et al*., 2020) for the Chelsa dataset, and also available at https://chelsa-climate.org.

### Ecological niche modelling

**Figure 1.**
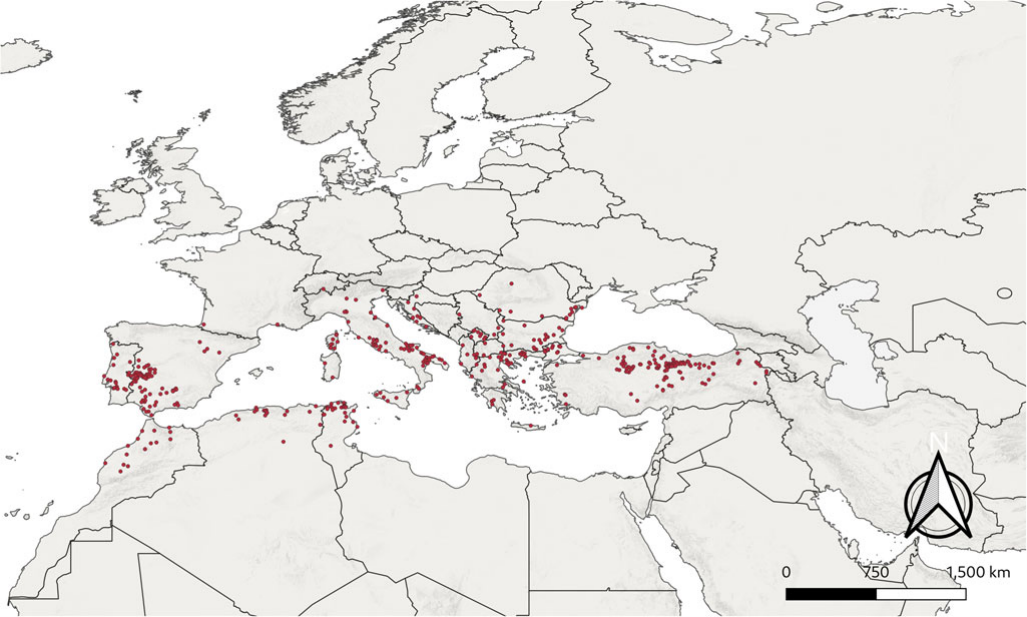
All occurrence points of *Hyalomma marginatum* after cleaning and thinning.

Before building the models, occurrence records that fell outside environmental raster pixels were removed. In order to reduce spatial autocorrelation, the locations for *H. marginatum* were thinned to a 5 km radius using the spThin package for R (Aiello-Lammens *et al*., 2015). The resulting occurrence data consisted of 470 locations (Fig. 1) which were split into a training set (70%: *n* = 335), a preliminary test set (cross-validation set) for evaluating the candidate models (25%: *n* = 111) and another secondary independent test set for evaluation of final models (5%: *n* = 24). To simulate the accessible M space (Soberon and Peterson, 2005; Barve *et al*., 2011) for *H. marginatum*, a buffered minimum convex polygon with a buffer area of 100 km around the occurrence records was created and environmental variable rasters representing the M space were clipped to this buffer area before building models. Thinning and the creation of the buffer zone were carried out with ellipsenm package for R, available at https://github.com/marlonecobos/ellipsenm (Cobos *et al*., 2020). Three sets were built by setting correlation thresholds between variables using the vifcor function in the R package usdm (Naimi, 2017). The vifcor function selects the variables with lower variance inflation factors (vif) from correlation pairs to build the sets. The correlation thresholds were 0.9, 0.8 and 0.75, respectively. For the first model dataset sets 2 and 3 were identical, so only set 1 and set 2 were used to build the models. For the Chelsa dataset some of the Bioclim+ variables are not available for future projections (vpd, hurs, rsds and cmi). Thus, 3 different sets were built not including these variables to build a separate second model (model 2) for future projections.

ENMs were built with the maximum entropy algorithm (Phillips and Schapire, 2004) using MaxEnt 3.4.4 (Steven *et al*., 2021) implemented in the Kuenm package (Cobos *et al*., 2019) for R. Using the Kuenm package, calibration models were built with all combinations of MaxEnt features L (linear), Q (quadratic), P (product) and H (hinge). All models were repeated with regularization multiplier parameters 0.1, 0.2, 0.3, 0.4, 0.5, 0.6, 0.7, 0.8, 0.9, 1, 2, 3, 4 and 5. The performance of all these models were evaluated primarily with the partial receiver operating characteristics (pROC) test and an omission threshold rate of 5% (Peterson and Soberón, 2012; Aguilar-Domínguez *et al*., 2021), secondary evaluations were done with Akaike information criterion corrected for small sample size (AICc). The selected eventual calibration models were statistically significant, that have omission rates below 5% and with ΔAICc values below 2 (Warren and Seifert, 2011; Nuñez-Penichet *et al*., 2021).

After model calibrations, final models were created with the selected parameters in the resulting calibration models using all the same occurrences of training and testing data with 10 bootstrap replicates. Then, these final models were additionally evaluated with the independent location data (*n* = 24) that were not used for building and selecting these models.

Map binarization was done by using the average logistic thresholds of maximum training sensitivity plus specificity of 10 replicates (Liu *et al*., 2013). Consensus maps of the niche models for the future projections were created by taking the average of GCMs. In order to show the effects of model projections under novel future conditions, we transferred models with allowed projections in novel climate conditions (extrapolation with clamping to the GCM scenarios) (Cobos *et al*., 2019). To see the places where conditions are more extreme compared to the calibration area of the models, a mobility-oriented parity metric (MOP) analysis was carried out and extrapolation risk of transfer regions was calculated with nearest 10% reference (Owens *et al*., 2013; Alkishe *et al*., 2020; Flores-López *et al*., 2022). Because of memory limitations, resolution of calibration area and transfer rasters were changed from 30 s to 2.5 min resolution and MOP analysis were carried out with the mop function included in the kuenm package (Cobos *et al*., 2019). Again, consensus maps for MOP were created taking the average of all GCMs for each ssp scenario and 4 MOP maps were created in total.

## Results

### Model parameters

Environmental parameters selected based on correlation thresholds and vif for variable sets used in model calibrations are shown in Table 1. In total 420 candidate models were created for the first model (model 1). All of 420 candidate models were statistically significant (pROC test *P* < 0.05); however, of these only 82 fulfilled the omission rate criteria of 5% (omission rate = 3.6%). Only one of these met the AICc criteria of ⩽2 (ΔAICc = 0.90). This final selected model is a linear quadratic model with a regularization multiplier parameter of 0.2, which uses variables included in set 1 with a mean area under the curve (AUC) ratio of 1.21. The final evaluation with the independent test set resulted in an omission rate of 8.3%. Although this value is larger than the 5% threshold set for evaluations with the cross-validation sets, the independent test set had a sample size of *n* = 24, and 8.3% omission rate means that the final model classified 22 of 24 test locations correctly which is a pretty good result for a machine learning algorithm. The percent contribution and permutation importance of 12 predictors are shown in Table 2. Out of these, the most important parameter was cmi with a contribution of 33.1%, followed by Bio14 (precipitation amount of the driest month) with a 24.2% contribution and rsds with an 8.4% contribution. Bio6 (minimum air temperature of the coldest month) was the fourth most important predictor (7% contribution).

**Table 1.** Environmental predictors used for different sets in model 1 and model 2 with explanation of the predictors in the first column

	Model 1		Model 2		
	Set 1	Set 2	Set 1	Set 2	Set 3
Bio3 (isothermality)	√	√	√	√	x
Bio4 (temperature seasonality)	√	x	√	x	x
Bio6 (min. air temp. of the coldest month)	√	x	√	x	x
Bio7 (annual range of air temperature)	√	√	√	√	√
Bio13 (precipitation amount of the wettest month)	√	√	√	√	√
Bio14 (precipitation amount of the driest month)	√	√	√	√	x
Bio15 (precipitation seasonality)	√	√	√	√	√
Gdd0 (growing degree days heat sum above 0°C)	x	x	√	x	x
Gst (mean temperature of the growing season)	√	√	√	√	√
Gsp (precipitation amount of the growing season)	√	x	√	x	x
Net primary productivity (npp)	√	x	√	x	x
Surface downwelling shortwave radiation (rsds)	√	x	x	x	x
Climate moisture index (cmi)	√	x	x	x	x

**Table 2.** Percent contribution of environmental predictors to model 1 and model 2

Model 1		Model 2	
Predictor	Percent contribution	Predictor	Percent contribution
Cmi	33.1	Bio13	31.3
Bio14	24.2	Bio14	19.4
Rsds	8.4	Gdd0	13
Bio6	7	Bio6	8.2
Bio15	6	Npp	8
Bio13	5.7	Bio15	7.5
Gst	4.2	Bio4	4.9
Npp	4.1	Bio3	4.5
Bio3	2.1	Gst	2.2
Bio7	1.8	Bio7	1.1
Bio4	1.8	Gsp	≈ 0
Gsp	1.5		

Out of 630 candidate models for the second Chelsa model (model 2) built for future projection, 625 were statistically significant (pROC test *P* < 0.05). Out of these, 201 met the 5% omission rate criteria (≈ 4.5%), and only 2 of them met the AICc criteria: a PH (product hinge) model with a regularization multiplier of 2 (ΔAICc ≈ 0), and a QPH (quadratic product hinge) model with a regularization multiplier of 2 (ΔAICc ≈ 1.27). Both of these final calibration models used the set 1 variables created for the second model. Mean AUC values were 1.23 and 1.20 for the product hinge and quadratic product hinge models, respectively. The final evaluation with the independent test set showed omission rates 8.3% and 12.5% for the PH and QPH models, respectively, indicating that the PH model is better for predictions. For the second model, there were 11 predictors. The biggest contributors were Bio13 (precipitation amount of wettest month) with 31.3% followed by Bio14 (19.4% contribution) and Gdd0 (growth degree days above 0 °C) with a 13% contribution. Like model 1, Bio6 was the fourth important predictor for model 2 with 8.2% contribution. Threshold values for creating binary maps were 0.409 and 0.483, respectively.

### Current and future predictions

**Figure 2.**
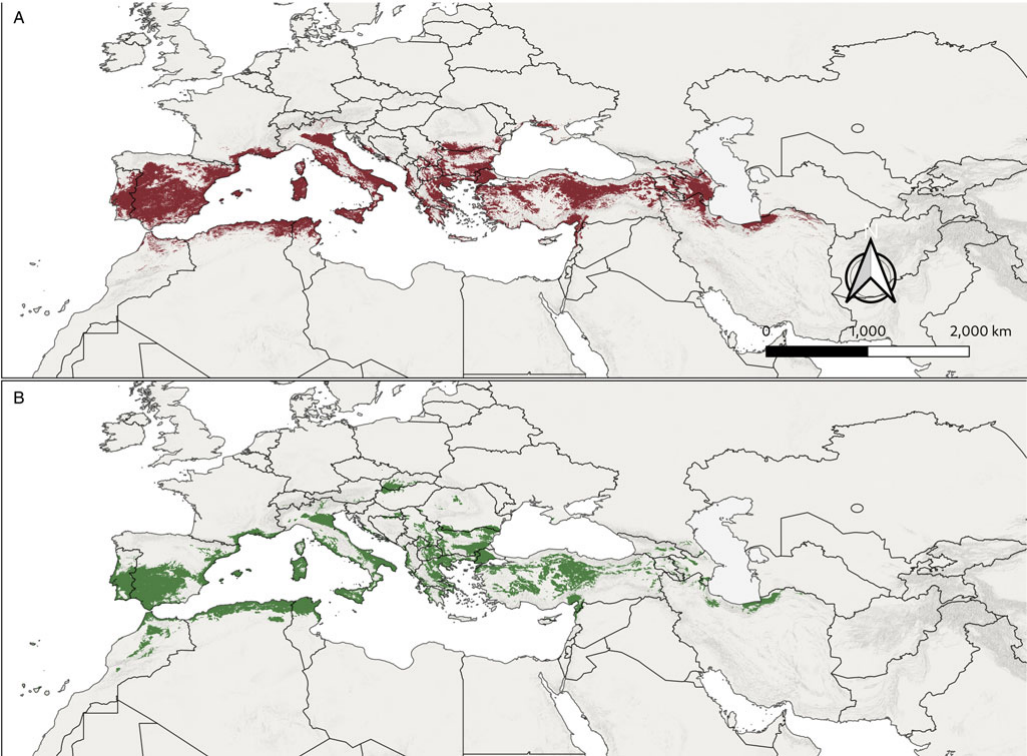
Maps of predicted suitable areas from the ENM results. (A) Red areas show the suitable regions under current conditions according to model 1. (B) Green areas show the suitable regions under current conditions for model 2.

Suitable areas for present conditions are shown in Fig. 2. According to the first model, the current climatically suitable region for *H. marginatum* stretches out from Iberian Peninsula to Anatolia and the Caspian Sea. This region includes most of the Mediterranean basin. This pattern mostly coincides with the reported locations of *H. marginatum.* The predictions of 2 models are mostly compatible with each other. Compared to the first model, the prediction of the second model for the current distribution shows a wider suitable area in some regions, especially in North Africa and near Hungary. Also, the first model shows wider suitable regions in Trans-Caucasus and Spain compared to the second model. This might be due to the fact that the second model does not include parameters like cmi and rsds which are present in the first model. On the contrary, the second model includes another important parameter, gdd0, which was eliminated from the first model in the correlation and vif selection procedure.

**Figure 3.**
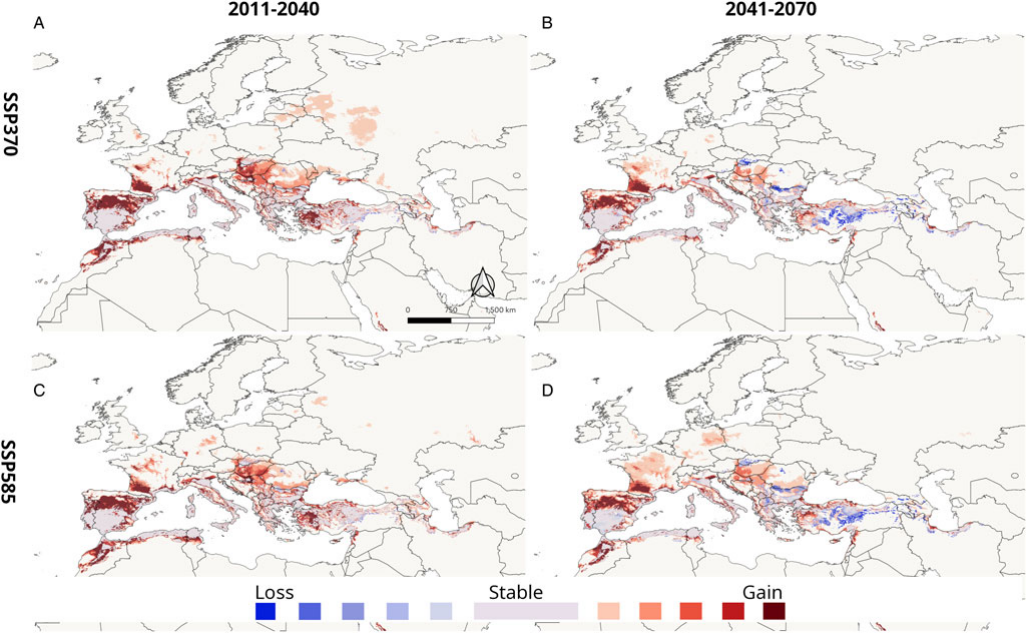
Maps of predicted suitable areas for future average of 5 GCM scenarios with differing degrees of loss and gain compared to current conditions for model 2. (A) For ssp370 in the 2011–2040 period. (B) For ssp370 in the 2041–2070 period (C) for ssp585 in the 2011–2040 period (D) for ssp585 in the 2041–2070 period.

**Figure 4.**
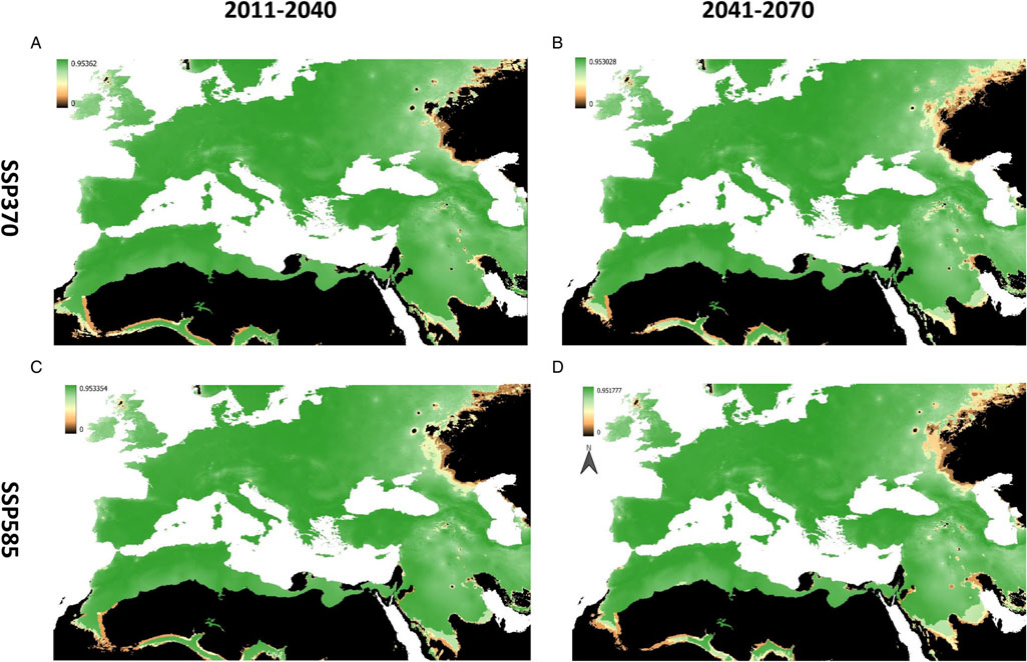
Extrapolation risk in 4 GCM projections of *H. marginatum* with MOP10%. Green to black scale shows increasing risk extrapolation where black areas are regions with strict extrapolation. (A) For ssp370 in the 2011–2040. (B) For ssp370 in the 2041–2070 period. (C) For ssp585 in the 2011–2040 period. (D) For ssp585 in the 2041–2070 period.

The predicted changes in suitable areas under GCM scenarios compared to present conditions are also shown in Fig. 3. Future projections under ssp370 scenarios for 2011–2040 show a significant widening of the climatically suitable area. The direction of the expansion is northwards in Europe, westwards in Anatolia and southwards in North African regions. Interestingly, the projections for the 2041–2070 period do not show such widening compared to the 2011–2040 period in eastern Europe and Anatolia; for example, the patches of new barely suitable regions in Baltic regions and Russia are lost in the 2041–2070 scenarios. In addition to that, there are significant declines in some regions in both ssp370 and ssp585 scenarios; these areas of suitability decline are especially distinct in central Anatolia and some areas in Balkans and central Europe. While both ssp370 and ssp585 show similar patterns in new areas for the 2041–2070 period, ssp585 scenarios present a wider new region of low suitability in France and eastern Germany. The results of MOP analysis done for between calibration area and future projections are shown in Fig. 4. High extrapolation risk areas are located relatively far from the current distribution and projected shifting areas of *H. marginatum* and the regions with high or strict extrapolation start from the north and east of Caspian Sea, Middle East (Arabian Peninsula, Israel and Egypt) and Africa (excluding northwest Africa).

## Discussion

The outputs from the first model show that the main climatic limiting factors for *H. marginatum* are cmi and precipitation, followed by surface radiation and minimum temperatures. Relative humidity or moisture is crucial for ticks, as they are highly susceptible to cuticular water loss in their off-host state (Knülle and Rudolph, 1982; Benoit and Denlinger, 2010; Estrada-Peña *et al*., 2012; Requena-García *et al*., 2017; Leal *et al*., 2020). The importance of water content of air for *H. marginatum* has also been demonstrated in previous habitat suitability models (Estrada-Peña *et al*., 2011; Fernández-Ruiz and Estrada-Peña, 2021). Another important parameter is incoming surface radiation, which is a relatively neglected parameter in ENMs for ticks despite the fact that it is a critical parameter for small arthropods (Davis *et al*., 2005; Battisti *et al*., 2013; Barrett and O'Donnell, 2023). Solar radiation might affect many different parameters related to ticks. Firstly, it is the main driver of microclimate temperature, and higher levels of solar radiation might increase tick abundance and questing activity (Kiewra *et al*., 2014; Del Fabbro *et al*., 2015). However, due to ultraviolet-B (UVB) radiation, very high levels of solar radiation might also have deleterious effects on small ectotherms like acari (Sakai *et al*., 2012; Sudo and Osakabe, 2015).

The predicted area for the current conditions in model 1 is mostly compatible with previous habitat suitability models created for *H. marginatum* (Williams *et al*., 2015; Fernández-Ruiz and Estrada-Peña, 2021). Additionally, the model output shows potential suitable areas in Trans-Caucasus and Iran, especially in northern Iran near the coast of the Caspian Sea, despite that the dataset of locations used for the models did not include any locations from this region. Although no exact coordinates are presented in the literature, CCHF cases and also *H. marginatum* ticks carrying the virus have been reported from this area (Shemshad *et al*., 2012; Sofizadeh *et al*., 2014; Telmadarraiy *et al*., 2015; Sedaghat *et al*., 2017).

Model 2 output shows a larger climatically suitable area compared to the more restrictive predictions of model 1. Important parameters like moisture index (cmi) and surface radiation (rsds) were left out for this model, as they are not available for future scenarios. Moisture index is replaced by precipitation of the wettest period (Bio13) as the most important parameter in model 2, which was in sixth place in the previous model. Although net precipitation amount is not as strong as water deficit or moisture index as a predictor, this shows the importance of the water balance for climatic suitability. In addition, surface radiation is replaced by gdd0 (growth degree days above 0 °C), which was eliminated from model 1 due to correlation threshold with other parameters. Fulfilling the required degree days is an important necessity for *H. marginatum* populations to establish in a new region (Estrada-Peña, 2023). It has been reported that a yearly accumulated temperature of 3000–4000 °C is necessary for this species (Estrada-Peña *et al*., 2011); a northern limit roughly coincides with 47°N.

With the increased temperature in recent decades, new reports of *H. marginatum* have also been increasing in Europe (Hornok and Horváth, 2012; Duscher *et al*., 2018; Bah *et al*., 2022; Lesiczka *et al*., 2022). Gillingham *et al*. (2023), who modelled the probability of survival and establishment of *H. marginatum* populations depending on cumulative degree days for the United Kingdom, forecasted increased risk in southern UK in the future. Also, the prediction maps for the climatically suitable area for ssp370 and ssp585 scenarios point to a northward expansion in Europe in the future. Depending on the models trained with trends in climate and tick records between 1970 and 2018, Fernández-Ruiz and Estrada-Peña (2021), assumed that the suitable climatic area in Europe would expand while maintaining the original Mediterranean distribution. Additionally, Williams *et al*. (2015) also predicted a northward expansion under AR5 climate scenarios in the future. Another parameter that should be considered is minimum temperatures, which occupied fourth place in both models 1 and 2. In addition to the above-mentioned effect of temperature (degree days) on development time, overwintering survival is another important factor for *H. marginatum*, as most adults overwinter in the field (Valcárcel *et al*., 2020). An increase in the minimum temperatures in the field might increase winter survival and contribute to the ongoing and possible future expansion in the northern limits of *H. marginatum* populations (Estrada-Peña *et al*., 2012). Projections for future distributions are compatible with these predictions, showing a very similar pattern of new suitable regions in North Spain, France, the Balkans and western Anatolia. The projections for 2041–2070 point to a continuation of this expansion; however, the projection also points to significant declines in some regions, mostly in central Anatolia and the Balkans. This is most probably due to the decrease in precipitation, as climatic simulations predict that the impact of decreased precipitation will be much higher in the eastern Mediterranean, especially in Turkey (Hoerling *et al*., 2012; Turkes *et al*., 2020). It was previously suggested that the northern limit for *H. marginatum* is thought to be mainly determined by temperature, while the southern limit is determined by precipitation and humidity (Gray *et al*., 2009), and expansion in the north and contraction in the south is an expected outcome in the future.

It also has to be considered that in some regions, the change in suitability might be more complex than this pattern. With projected ongoing climate change, an interchange of climatically suitable and unsuitable areas can be seen in models (Gillingham *et al*., 2023). A previous model using RCP8.5 scenarios carried out locally for Romania depicted an increase in suitable areas until 2050, followed by a decreasing suitability until 2070 (Domşa *et al*., 2016), which was also detected by future predictions in the current study (Fig. 3). This rather complex pattern in suitability might be due to the 2 main factors (temperature and precipitation) determining suitability. For instance, present-day models show a highly suitable region for *H. marginatum* in the central and central-north parts of Turkey. Contrasting with the relatively more northward expansion in Europe, future projections indicate that after 2040, this suitable area might shift towards coastal areas where required precipitation would be available compared to the now more arid continental regions.

Assessing the potential distribution range of ticks is important to predict the risk of emergence and re-emergence of tick-borne diseases (Estrada-Peña, 2008; Zhao *et al*., 2021). In this sense, the interaction between ticks and climate is extremely important, as the survival and biological functions of ticks depend strongly on the external micro-abiotic environment (Gray *et al*., 2009). It should also be kept in mind that finding a suitable host is one of the most important drivers, which affects the distribution of tick species in an area. On the contrary, *H. marginatum* is a 2-host species and uses a wide range of vertebrates at different stages of its life cycle; this complex behaviour of this species is another reason for the difficulties in predicting the future distributions of these vectors (Apanaskevich and Horak, 2008; Valcárcel *et al*., 2020; Bonnet *et al*., 2022). For instance, an area might be classified as suitable for a species by an ENM in future, but the absence or scarce distribution of suitable hosts would prevent the establishment of populations in the area. Conversely, unexpected novel hosts in new environments would provide the necessary link for dispersal in new environments (Bakkes *et al*., 2021). Additionally, landscape cover, configuration and habitat fragmentation are other potential factors on the distribution of ticks and transmission of tick-borne diseases (Suzán *et al*., 2012; Perez *et al*., 2016). A previous study showed that in Turkey, the number of CCHF cases was associated with high landscape fragmentation and connectivity (Estrada-Pena *et al*., 2010). On the contrary, Bah *et al*. (2022) showed that in southern France, sclerophyllous vegetated or sparsely vegetated open natural areas present favourable habitats for *H. marginatum* rather than more humid or urbanized areas, a similar pattern is also present in Anatolia where the highest distribution is in the central-north while *H. marginatum* is significantly absent both in humid and densely forested Black Sea coast and hot and dry southern regions. Ecological niche models are generally more deterministic models that build the representative fundamental niche using presently available abiotic parameters and locations, then project this assumed niche space to assumed future abiotic parameter data calculated by predictive global circulation simulations (Escobar, 2020; Sillero *et al*., 2021). Thus, these projections are for predicting the climatically suitable areas in the predicted future under assumed scenarios. However, these kinds of models are among the most useful tools presently available for estimating the potential risk of vector-borne diseases and deciding the necessary precautions (Medlock and Leach, 2015, Ortega-Guzmán *et al*., 2022). MOP analysis is one of the tools to interpret the potential risk of uncertainty for transfer of models to novel conditions (Owens *et al*., 2013; Cobos *et al*., 2019). MOP analysis showed that the current and predicted future distribution areas did not include areas with high risk of extrapolation. Uncertainty maps for *H. marginatum* are similar to the Europe and North Africa projections of a previous uncertainty analysis of worldwide projections of the distribution of another tick species (*Rhipicephalus sanguineus*) to RCP scenarios done by Alkishe *et al*. (2020). The main difference is that the present MOP results of projections to ssp scenarios show new and wider areas with high extrapolation in North Africa and Middle East compared to the previous paper by Alkishe *et al*. (2020).

With the ongoing human-induced changes in the biotic and abiotic environment we have been observing a significant increase in dispersal and abundance of *H. marginatum* and also an increase in the transmission of pathogens carried by *H. marginatum*. Future distribution projections indicate a significant increase in potential risk due to these factors, so it is important to build both statistical and process-based mechanistic models with new data. Also publishing coordinates is strongly suggested, as it is observed that there are many studies which report species presence without any coordinates that can help future coordination between researchers and provide more reliable models. As *H. marginatum* is the vector of CCHF, change in predicted distributional range might point out potential new risk areas or widening of current endemic areas. This study may be helpful to forecast new risk areas and therefore to expand awareness and to start well-adapted prevention strategies against CCHF in these areas. Similarly, our models show that the climatically suitable region for *H. marginatum* matches the current distributional area, which can be interpreted as the need to strengthen and to maintain control measures to CCHF in the future.

## Data Availability

Locations used in this study are available at https://data.mendeley.com/datasets/n8b3kps7nk/1.
